# AI-driven audience clustering in sport media: a human–computer interaction approach using ‘CoPE-DEC’

**DOI:** 10.3389/fncom.2026.1767724

**Published:** 2026-01-29

**Authors:** Yong-Seok Jang

**Affiliations:** Department of Sport Media, Kyung Hee University, Yongin-si, Gyeonggi-do, Republic of Korea

**Keywords:** artificial intelligence, CoPE-DEC technology, deep learning, human–computer interaction, sports media viewer characteristics

## Abstract

This study investigates the characteristics and underlying patterns of sports media audiences from a human–computer interaction (HCI) perspective using artificial intelligence–based deep learning analysis, with the aim of providing foundational data for the sports media industry. To this end, a novel unsupervised clustering framework, the Column-conditioned Prototype-Enhanced Deep Embedded Clustering (CoPE-DEC) technique, was employed to model and analyze multidimensional viewer experience data derived from sports media consumption contexts. The analysis identified three distinct audience clusters with differentiated behavioral, attitudinal, and value-oriented characteristics. The first cluster, labeled “Sports Value Orientation,” was characterized by enhanced concentration during sports viewing, promotion of cooperative skills, motivation for health and exercise, vicarious satisfaction, aesthetic appreciation of sports movements, and admiration for athletes’ professional and economic success. The second cluster, termed “Sports Consumption Culture Orientation,” exhibited a strong preference for sports broadcasts over entertainment content, frequent consumption of online sports media, active engagement with preferred sports, participation in sports-related tourism and activities, acquisition of sports skills through media, and consumption of sports-related products. The third cluster, identified as “Sports Attitude Orientation,” reflected predominantly social and emotional dimensions of sports viewing, including improved social adaptation, relationship formation, group cohesion, stress relief, psychological stabilization, healthy competitive attitudes, and enhanced overall wellbeing. These findings demonstrate that AI-driven deep learning approaches, particularly the CoPE-DEC framework, are effective in uncovering latent audience typologies and preference structures in sports media consumption environments. By integrating HCI principles with advanced clustering techniques, this study offers a methodological contribution to audience analysis research and provides practical implications for audience segmentation, personalized content design, and strategic decision-making in the sports media industry. Future research is encouraged to extend this approach by incorporating diverse AI methodologies and multimodal data sources to further advance interdisciplinary insights at the intersection of HCI, artificial intelligence, and sports media studies.

## 1 Introduction

As digital transformation accelerates across media ecosystems, understanding user behavior and experience has become a central challenge in both academia and industry. In this context, Human–Computer Interaction (HCI) has emerged as a core disciplinary framework for explaining how users perceive, interpret, and engage with digital systems, providing theoretical and methodological foundations for user-centered design and experience optimization (Card et al., 1983; Norman, 2013; Preece et al., 2022). Recent advances in artificial intelligence (AI), particularly deep learning, have further expanded the analytical capacity of HCI research by enabling the modeling of complex, high-dimensional user data and uncovering latent behavioral patterns that are difficult to capture using traditional approaches.

The convergence of HCI and deep learning–based clustering techniques is especially meaningful in contemporary media environments characterized by hyper-personalization, platform diversification, and continuous user interaction. Unlike conventional analytic frameworks that rely on predefined categories or linear assumptions, representation-based deep clustering methods allow researchers to identify emergent user typologies directly from data, thereby supporting more precise and adaptive user segmentation (Xie et al., 2016; Li X. et al., 2022; Saito and Yamamoto, 2023). Among these approaches, Column-conditioned Prototype-Enhanced Deep Embedded Clustering (CoPE-DEC) offers a promising framework by integrating item-aware embeddings, prototype alignment, contrastive learning, and DEC-based refinement, enabling both interpretability and structural clarity in unsupervised clustering tasks.

This methodological advancement is particularly valuable in the sports media industry, where audience behavior has become increasingly complex and heterogeneous. The contemporary sports media environment is shaped by the convergence of over-the-top (OTT) platforms, interactive live streaming, real-time data-driven commentary, and immersive technologies such as augmented and virtual reality (AR/VR) (Funk et al., 2018; Ratten, 2021). Viewers are no longer passive recipients of broadcast content; rather, they actively engage with sports media across multiple platforms, participate in online communities, generate user-created content, and interact with athletes, teams, and other fans in real time. As a result, audience experiences are formed through dynamic interactions between human users and computational systems, making an HCI-oriented analytical perspective indispensable.

Recent trends in sports consumption—particularly among the MZ generation—further underscore the limitations of traditional analytical models. Contemporary audiences increasingly favor immediacy (real-time and short-form content), identity expression (community participation and social signaling), and reciprocal interaction (comments, voting, memes, and challenges) (Wohn and Freeman, 2020; Lim and Park, 2023). However, existing research on sports media audiences has largely relied on regression-based models, satisfaction–intention frameworks, or psychologically driven causal analyses that assume linear relationships and predefined constructs (Kim and Hwang, 2019; Lim and Lim, 2024). While these approaches offer valuable insights into specific relationships, they face structural constraints in capturing high-dimensional, nonlinear, and heterogeneous audience patterns across platforms and interaction contexts.

Moreover, prior studies often focus on *post hoc* explanations of viewing satisfaction or continued usage intention, rather than on the ex-ante identification and generalization of audience typologies. This limitation has become increasingly problematic as sports media data environments grow more complex, incorporating multimodal and high-frequency data from live broadcasts, OTT services, social media interactions, and search behaviors. In such environments, viewer behavior exhibits nonlinearity, temporal dynamics, and network diffusion effects that exceed the explanatory capacity of conventional models (Bunker and Susnjak, 2022; Horvat and Job, 2020).

Against this backdrop, we identify three critical limitations in existing approaches. First, many studies rely on regression-based or psychologically grounded models that presuppose linearity and stable variable relationships. Second, these methods struggle to capture the multidimensional and heterogeneous nature of contemporary sports media audiences. Third, there remains a lack of HCI-based, unsupervised, representation-driven audience typology frameworks capable of generalizing viewer characteristics across platforms and interaction contexts. These gaps point to the need for advanced AI-driven methodologies that can model audience behavior more holistically and flexibly.

To address these limitations, the present study applies the Column-conditioned Prototype-Enhanced Deep Embedded Clustering (CoPE-DEC) framework to sports media audience analysis from an HCI perspective. CoPE-DEC directly responds to the identified gaps by (1) employing column-conditioned embeddings to preserve item-specific semantics, (2) enhancing interpretability through prototype alignment, and (3) improving cluster sharpness and stability via contrastive learning and DEC fine-tuning. By leveraging these mechanisms, the proposed approach enables the extraction of latent audience typologies that reflect not only behavioral patterns but also value orientations and attitudinal dimensions of sports media consumption.

Accordingly, the objective of this study is to predict and classify sports media viewer characteristics using an AI-based deep learning framework grounded in HCI principles. By segmenting audiences based on empirical viewing behavior, this study aims to provide foundational data for market segmentation, personalized content design, and strategic decision-making in the sports media industry. Furthermore, the study seeks to contribute to the development of an AI-based knowledge system for predictive sports media research and to establish an integrated benchmark for evaluating both cultural relevance and market efficiency through data-driven audience classification.

## 2 Theoretical background

### 2.1 Sports media viewing behavior theory

Sports media viewing behavior, particularly in live streaming environments, has been extensively examined through multiple theoretical perspectives, most notably Uses and Gratifications Theory (UGT), Flow Theory, and Parasocial Interaction Theory. These frameworks provide complementary explanations for why and how audiences engage with sports media content in increasingly interactive and technology-mediated contexts.

Uses and Gratifications Theory conceptualizes media users as active and goal-oriented agents who selectively engage with media to satisfy specific psychological, social, and informational needs rather than as passive recipients of content (Katz et al., 1973). In contemporary sports media environments, this perspective has been extended to explain viewers’ motivations for consuming live sports streams, including entertainment, real-time information acquisition, social interaction through chat functions and online communities, and emotional engagement with teams and athletes (Sjöblom et al., 2019; Lim and Park, 2023). Recent studies further suggest that the interactivity embedded in digital sports platforms enhances users’ perceived autonomy and involvement, reinforcing active media selection and sustained engagement (Wohn and Freeman, 2020).

Advancements in real-time feedback mechanisms—such as live comments, emojis, and audience reactions—have transformed sports streaming platforms into collective emotional spaces, where viewers experience shared excitement, solidarity, and collective cheering. These developments align closely with Flow Theory, which explains the immersive psychological state experienced when individuals perceive an optimal balance between challenge, skill, and engagement (Csikszentmihalyi, 1990). In sports live streaming contexts, flow emerges when high-quality audiovisual content, real-time interaction, and emotionally charged competition converge, leading to temporal distortion, deep concentration, and heightened affective involvement (Hamari and Sjöblom, 2017). Empirical research indicates that such flow experiences significantly increase re-watching intentions, viewing duration, and long-term platform loyalty (Chen et al., 2022).

Parasocial Interaction Theory offers an additional explanatory lens by focusing on the quasi-social relationships that viewers develop with media figures, commentators, or streamers, whom they perceive as familiar and emotionally accessible despite the absence of reciprocal interaction (Horton and Wohl, 1956). In sports streaming environments, parasocial bonds are strengthened through commentators’ narrative styles, humor, empathy, expertise, and nonverbal expressions, fostering emotional intimacy and trust (Frederick et al., 2022). Recent studies demonstrate that parasocial interaction plays a critical role in shaping viewer satisfaction, perceived credibility, and continued engagement, including long-term subscription behavior and community participation (Tukachinsky, 2021; Wohn and Freeman, 2020).

From a broader social and cultural perspective, engagement with sports media content influences viewers’ socialization processes and emotional development. Sports viewing has been shown to promote cooperative values, respect for rules, social cohesion, and collective identity formation, although these effects may vary depending on the values and norms conveyed through media representations of sport (Funk et al., 2018; Ratten, 2021). Sports consumption is also deeply embedded within contemporary consumer culture, functioning as a symbolic and identity-driven practice that reflects broader social meanings, lifestyle aspirations, and group affiliations (Belk, 2013; Giulianotti, 2022). In this sense, sports media consumption extends beyond leisure activity to participate in the reproduction of cultural norms and market-oriented structures within society.

Recent empirical research has further classified sports consumption culture into multiple domains, including media-based information consumption, sport-related product purchases, experiential participation such as tourism and events, and digitally mediated skill acquisition (Zhang et al., 2021; Kim et al., 2024). Identifying distinct viewer types within this framework enables a more nuanced understanding of differences in preferences, motivations, and behavioral patterns across sports media audiences. Sports values and attitudes are thus conceptualized as expectation-based cognitive and affective orientations, shaped by prior experiences, social influence, and informational cues provided by media platforms (Oliver, 2014; Chiu et al., 2017).

Taken together, these theoretical perspectives indicate that the study of sports media viewing behavior extends beyond explaining satisfaction with isolated media products or services. Instead, sports media consumer theory functions as a strategic analytical framework for understanding long-term engagement, loyalty formation, and value co-creation in interactive media environments. By integrating motivational, psychological, social, and cultural dimensions, contemporary sports media research provides critical insights for enhancing user experience design, strengthening fan relationships, and sustaining competitive advantage in rapidly evolving digital sports ecosystems (Zeithaml et al., 2020; Pizzo et al., 2023).

### 2.2 Classification of characteristics of sport media content viewers

Market segmentation has long been recognized as a fundamental strategy for understanding heterogeneous consumer populations and for designing effective marketing and communication strategies, particularly in highly competitive industries such as sports. Classical marketing literature conceptualized segmentation as the process of dividing markets into relatively homogeneous groups based on shared characteristics, including demographic, geographic, psychographic, and behavioral variables (Kotler and Keller, 2022). In the context of sports consumption, these variables have traditionally been used to explain differences in attendance, media usage, merchandize purchasing, and fan loyalty.

Early segmentation studies in sports marketing primarily relied on demographic or single-dimension behavioral indicators, such as age, gender, income, or frequency of consumption. However, subsequent research has demonstrated that such approaches provide only limited explanatory power, as sports consumers often exhibit complex combinations of motivations, values, lifestyles, and engagement patterns that transcend demographic boundaries (Funk et al., 2016; Yoshida et al., 2017). As a result, contemporary segmentation research increasingly emphasizes the integration of psychological, attitudinal, and behavioral variables to capture the multidimensional nature of sports media audiences (Trail et al., 2019).

With the rapid digitization of sports media and the proliferation of OTT platforms, social media, and interactive live streaming services, the characteristics of sports media viewers have become more fragmented and dynamic. Viewers engage with sports content across multiple platforms, exhibit varying levels of emotional involvement and social interaction, and differ substantially in their consumption of related products and experiences (Ratten, 2021; Lim and Park, 2023). Consequently, no single segmentation variable can be considered universally superior; rather, the selection of segmentation criteria must align with the analytical objective and the media context in which consumption occurs (Wedel and Kannan, 2016).

In parallel with these conceptual developments, research on predicting sports media viewership has evolved from qualitative, experience-based forecasting toward quantitative and data-driven approaches. While qualitative methods—drawing on expert judgment from broadcasters, league officials, and event organizers—remain valuable for contextual interpretation, their reliance on subjective assessment limits scalability and predictive precision in rapidly changing media environments. The accumulation of large-scale digital trace data, including viewing logs, interaction records, and platform analytics, has highlighted the advantages of quantitative modeling in reducing forecast uncertainty and improving generalizability (Chae and Kim, 2021; Popp et al., 2020).

Recent scholarship has increasingly applied machine learning and deep learning techniques to sports-related prediction and segmentation tasks, including audience clustering, engagement prediction, and consumption pattern analysis. These approaches are particularly well suited to handling high-dimensional, nonlinear, and heterogeneous data structures that characterize contemporary sports media environments (Horvat and Job, 2020; Bunker and Susnjak, 2022). Deep learning–based models enable the extraction of latent representations of consumer behavior, allowing for the identification of viewer segments that reflect underlying value orientations, participation patterns, and attitudinal tendencies rather than surface-level characteristics alone (Chen, 2024; Li X. et al., 2022).

From a sports media perspective, AI-driven audience classification offers several strategic advantages. First, it supports proactive estimation of viewer preferences prior to content release or event broadcasting. Second, it facilitates the empirical identification of diffusion pathways and ripple effects across platforms and communities. Third, it enables differentiated content design, targeted marketing, and personalized interface development tailored to distinct viewer segments. By complementing the limitations of traditional qualitative and rule-based segmentation approaches, AI-based classification provides a systematic and scalable foundation for understanding sports consumers’ decision-making processes in digital environments.

Taken together, recent advances in market segmentation theory and AI-based analytics underscore the necessity of adopting integrated, data-driven frameworks for classifying sports media content viewers. Such frameworks are essential not only for academic understanding of audience heterogeneity but also for practical applications aimed at revitalizing the sports media industry, enhancing fan engagement, and informing evidence-based policy and marketing strategies in an increasingly competitive and technology-driven marketplace.

## 3 Research questions

Building upon the theoretical background and the literature review on sports media consumption and audience behavior, this study seeks to address the following research questions:

Research Question 1 (RQ1): How can sports media audiences be segmented using deep learning-based analytical methods?

Research Question 2 (RQ2): What are the distinctive characteristics of the audience groups identified through deep learning-based segmentation?

These questions aim to explore both the methodological approach to segmenting sports media audiences and the behavioral, attitudinal, and cultural characteristics that differentiate the resulting clusters. By addressing these questions, the study intends to provide actionable insights for sports media management, marketing strategies, and the application of artificial intelligence in audience analysis.

## 4 Methods

### 4.1 Data collection and analysis

The data for this study were obtained from the publicly available dataset, “Sports Live Streamer: Viewer Experience Survey Data,” hosted on the Mendeley Data platform. This dataset consists of survey-based behavioral and cognitive measures collected from a large number of sports content consumers, including viewers of YouTube streams, sports reactions, sports analysis, and sports broadcast jockeys (BJs). Unlike simple viewing his-tory logs, the dataset encompasses a wide range of psychological, cognitive, attitudinal, and behavioral factors, making it particularly suitable for research on sports media consumption, consumer behavior, and fandom.

For this study, the dataset was utilized as the primary source to analyze sports media audiences. The data were processed and analyzed using Column-conditioned Proto-type-Enhanced Deep Embedded Clustering (CoPE-DEC), an artificial intelligence-based deep learning method. This approach enabled the extraction of latent audience patterns and characteristics, providing a robust foundation for segmenting viewers based on multidimensional behavioral and cognitive attributes.

### 4.2 Variables and measures

The dataset employed in this study was designed to comprehensively capture the psychological and behavioral characteristics of sports live streaming users. Most items were assessed using a 5-point Likert scale (1 = not at all, 5 = very much). The key variables in this study included streamer image, flow (immersion), satisfaction, rewatching intention, and demographic characteristics. Streamer Image measures users’ perceptions of a specific sports streamer. It encompasses dimensions such as attractiveness, expertise, credibility, warmth, and quality of commentary. These factors are critical predictors of viewers’ attitudinal and behavioral responses, including trust, liking, and rewatching intention. In sports media, expert commentary serves as an essential criterion for evaluating content quality.

Flow captures the psychological experiences of users while engaging with live streaming content. This construct includes levels of immersion, time distortion, concentration, psychological engagement, and hedonic enjoyment. Sports live streaming is particularly conducive to flow due to its dynamic and real-time interactive nature. High flow experiences are significant predictors of viewer retention and positive attitudes toward the content. Satisfaction represents viewers’ overall evaluation of both the streamer and the con-tent. It was measured through satisfaction with the content, satisfaction with the streamer, appropriateness of delivery, and suitability of the live environment. Satisfaction is a pivot-al variable in media research, directly influencing repeated usage behavior and loyalty, and plays a similar role in sports streaming contexts.

Rewatching Intention refers to the users’ intention to rewatch the same streamer’s live broadcast, explore additional content from the streamer, and recommend the streamer to others via word-of-mouth. These indicators serve as key measures of behavioral persistence and loyalty within online media environments. Demographic Characteristics included gender, age, level of sports interest, primary viewing platform (e.g., YouTube, AfreecaTV, Twitch, Tving), viewing frequency, viewing time zone, and preferred content type. These variables can be used to examine differences across segments or serve as control variables in analytical models, thereby enhancing the explanatory power of the research framework. Overall, the dataset allows for a multifaceted analysis of psychological and behavior-al responses to sports live streaming. It is suitable for advanced statistical techniques, including structural equation modeling (SEM) and mediation and moderation analyses. Moreover, as the data were collected from Korean sports viewers, they accurately reflect the domestic sports media and streamer environment, providing valuable insights for plat-form strategy development, content format comparisons, and user experience research.

### 4.3 AI deep learning ‘CoPE-DEC’ data processing process

This study proposes an unsupervised clustering pipeline, Column-conditioned Prototype-Enhanced Deep Embedded Clustering (CoPE-DEC), designed for survey items where all variables are categorical. The pipeline integrates item-context-aware embedding modulation, prototype alignment, contrastive learning, and DEC fine-tuning. Given that all responses are categorical, identifier-like columns with high unique-value ratios and constant columns were removed. Additionally, items with responses representing less than 1% of the total respondents or with fewer than three occurrences were consolidated into a single sparse-response category to avoid an increase in dimensionality and model complexity during training.

To facilitate the analysis of respondents’ answer flow across items, response options were organized for each question like a dictionary, and sequential numerical identifiers were assigned to each option. The same word appearing in different items was assigned distinct identifiers to preserve item-specific context. These numbered sequences were then input into the embedding and clustering stages of the model, allowing for pattern tracking while maintaining context distinctions.

The model first constructs column-conditioned embeddings (Randrianantoanina, 2022). For each item, a category embedding matrix E_c∈R^(d_b) is established, and a FiLM modulator Φ:R^(d_b) → R^(2d_b) generates modulation parameters as defined in Equation 1. For the raw embedding of sample ie_((i,c)) = E_c [y_((i,c))] the column-conditioned modulation is then defined as in Equation 2.


(1) $$\left(\gamma_{c},\beta_{c})=\Phi (u_{c}),\;\gamma_{c}=\tanh(\gamma_{c}\right)$$

(2) $${\tilde{e}}_{i,c}=(1+\gamma_{c})⊙e_{i,c}+\beta_{c}$$

Each token h_((i,c)) is obtained by projecting onto a shared latent dimension d_m mthrough W_c:R^(d_m × d_b), such that h_((i,c)) = W_c e˜ _((i,c)). The tokens are then concatenated to form H_i = [h_((i,1)); …””;h_((i,C))]∈ R^(C × d_m), vectorized (with dropout), and passed through the MLP encoder f_θ yield the latent representation, as defined in Equation 3 (Lijing et al., 2022). Subsequently, each item is associated with an independent classification decoder g_(ϕ,c):R^d → R^(∣ν_c∣), which produces a predictive distribution using the softmax function, as described in Equation 4. From a theoretical perspective, the use of a softmax-based decoder ensures that each column-specific prediction forms a valid probability distribution, enabling stable gradient propagation and facilitating alignment between latent representations and column-level semantics. This design choice is particularly important in the proposed column-conditioned framework, as it allows each feature group to retain semantic independence while sharing a common latent embedding space. In addition to theoretical considerations, we conducted preliminary sensitivity analyses during the pilot experimentation phase to examine the robustness of the decoder-related hyperparameters. The results indicated that moderate variations in the decoder configuration did not lead to substantial changes in clustering stability or assignment consistency. This suggests that the proposed parameter settings are not overly sensitive and provide a reliable operating range for model optimization.


(3) $$z_{i}=f_{\theta }(\mathrm{vec}(H_{i}))\in {\mathbb{R}}^{d}$$

(4) $$\text{softmax}(g_{\phi ,c}(z_{i}))$$

To enhance interpretability and separability, pairwise latent prototypes are defined for each (item, category) pair. These prototypes are formulated by projecting the base embeddings into the latent space, such that P_((c,v)) = P_c E_c [v]∈R^d. The response-based mean prototype for sample i, as defined in Equation 5, serves as an interpretative reference point for the latent representation, guiding the alignment with z_i.


(5) $${\bar{P}}_{i}=\frac{1}{C}\sum_{c=1}^{C}P_{c,}y_{i,c}$$

The proposed model jointly optimizes noise reconstruction and prototype alignment without requiring labels. Data augmentation is implemented in a straightforward manner: some responses are intentionally masked, and, occasionally, responses are swapped across columns within the same batch. Under this scheme, the model is required to estimate the masked values using only the remaining item information, thereby learning inter-item relationships autonomously (Le Besnerais, 2008).

By attempting to reconstruct these corrupted samples, the model naturally captures the statistical dependencies among items. Specifically, the cross-entropy between the predicted distribution pˆ _((i,c)) at the masked positions and the original response y_((i,c))^”orig” “is computed. The mask reconstruction loss, as defined in Equation 6, is then obtained by averaging this cross-entropy over all positions where the mask indicator m_((i,c)) = 1.


(6) $$L_{\text{mask}}=\frac{1}{\sum_{i,c}m_{i,c}}\sum_{i=1}^{N}\sum_{c=1}^{C}m_{i,c}\mathrm{CE}({\hat{p}}_{i,c},y_{i,c}^{\text{orig}})$$

However, since some mini-batches may have few randomly masked responses, we stabilize convergence by using a global restoration term that faintly adds cross-entropy to all positions (Busoniu et al., 2010). This term is simply calculated as the average across all positions, as in Equation 7.


(7) $$L_{\text{full}}=\frac{1}{\mathrm{NC}}\sum_{i=1}^{N}\sum_{c=1}^{C}\mathrm{CE}({\hat{p}}_{i,c},y_{i,c}^{\text{orig}})$$

In addition, each item-choice combination is assigned a representative vector (prototype) in the latent space, and the average p ®_i of the prototypes of the choices actually selected by a person is regarded as that person’s typical response pattern. The prototype alignment loss, which induces the latent vector z_i generated by the encoder to be close to this typical pattern, establishes an interpretable reference point in the latent space as Equation 8 and sharpens the cluster centers. In short, the structure between items is learned through a restoration task that scores only some responses, a shallow overall restoration is added to ensure that the learning signal is not interrupted even in layouts with rare occlusion, and the latent representation is aligned to the response-based representative pattern to simultaneously secure separability and interpretability (Hang et al., 2020). In actual optimization, these three terms are combined with appropriate weights and minimized simultaneously.


(8) $$L_{\text{proto}}=\frac{1}{N}\sum_{i=1}^{N}{\left\|z_{i}-{\bar{p}}_{i}\right\|}_{2}^{2}$$

In the representation learning phase, we learn to distinguish between two augmented views of the same sample, labeling them as positive pairs and other samples in the same batch as negatives. The contrast objective used here is the InfoNCE loss, which increases the cosine similarity between the two latent vectors z_i^((a)) and z_i^((a)) of sample i while decreasing their similarity with other samples (Yiwei et al.). The scale is controlled by the temperature hyperparameter T, and the final form is expressed as Equation 9. In Equation 9, sim is the cosine similarity, arranging individuals with similar features closer together and individuals with distant features further apart to align the latent space.


(9) $$L_{\text{contr}}=\frac{1}{2N}\sum_{i=1}^{N}[\begin{array}{l} \log\frac{\exp(\frac{\mathrm{sim}(z_{i}^{\left(a\right)},z_{i}^{\left(b\right)})}{T})}{\sum_{j=1}^{N}\exp(\frac{\mathrm{sim}(z_{i}^{\left(a\right)},z_{i}^{\left(b\right)})}{T})}+\\ \log\frac{\exp(\frac{\mathrm{sim}(z_{i}^{\left(b\right)},z_{i}^{\left(a\right)})}{T})}{\sum_{j=1}^{N}\exp(\frac{\mathrm{sim}(z_{i}^{\left(b\right)},z_{i}^{\left(a\right)})}{T})} \end{array}]$$

This contrastive loss is combined with the previously described mask restoration, full restoration, and prototype alignment to form a single pre-training objective. The weights are adjusted appropriately, but are generally set to 1.0 for mask restoration, 0.3 for full restoration, 0.5 for prototype alignment, and 0.5 for contrastive learning, as shown in Equation 10:


(10) $$L_{\mathrm{pre}}=\lambda_{\text{mask}}L_{\text{mask}}+\lambda_{\text{full}}L_{\text{full}}+\lambda_{\text{proto}}L_{\text{proto}}+\lambda_{\text{contr}}L_{\text{contr}}$$

After initially selecting the pre-trained representation z_i using K-means, we distinguish the cluster boundaries using DEC receiver tuning (Lin et al., 2020). Then, we first assemble the interesting z_i that each individual belongs to each cluster base station μ_k (to indicate the check-in of the boundary), and then create a target group p_ik that further “sharpens” the interesting q_ik to reduce the difference between the two groups, and update the centers together. The interesting q_ik is defined in the same way as Equation 11, which surpasses Student-t.

After initially segmenting the latent representation z_i obtained through pre-training using K-means, the cluster boundaries are refined using DEC fine-tuning (Lin et al., 2020). Then, the soft probability z_i that each sample belongs to each cluster center μ_k is first calculated (reflecting the ambiguity near the boundary), and then the target distribution p_ik is created by further “sharpening” the probability, and the encoder and centers are jointly updated to reduce the difference between the two distributions. The soft probability is defined using the Student-t kernel as shown in Equation 11.


(11) $$q_{\mathrm{ik}}=\frac{{\left(1+\frac{{\left\|z_{i}-u_{k^{\prime }}\right\|}^{2}}{\alpha }\right)}^{-\frac{\alpha +1}{2}}}{\sum_{k^{\prime }=1}^{K}{\left(1+\frac{{\left\|z_{i}-u_{k^{\prime }}\right\|}^{2}}{\alpha }\right)}^{-\frac{\alpha +1}{2}}}$$

Here, the target distribution is constructed as in Equation 12 for sharpening, which makes the already large samples larger and the small samples smaller, and then the encoder and center are jointly updated as in Equation 13 to minimize the KL divergence (Finlayson, 2000).


(12) $$p_{\mathrm{ik}}=\frac{\frac{q_{ik^{\prime }}^{2}}{\sum_{i}q_{\mathrm{ik}}}}{\frac{\sum_{k^{\prime }}q_{ik^{\prime }}^{2}}{\sum_{i}q_{ik^{\prime }}}}$$

(13) $$L_{\mathrm{DEC}}=\sum_{i=1}^{N}\sum_{k=1}^{K}p_{\mathrm{ik}}\log\frac{p_{\mathrm{ik}}}{q_{\mathrm{ik}}}$$

Intuitively, this process involves rearranging the latent space so that the “soft probabilities q-computed by the model” are aligned with the “more confident targets p.” As a result, similar patterns become more tightly clustered, while dissimilar patterns move farther apart, sharpening the cluster boundaries. Once training converges, the final label for each sample is determined as kˆ_i = arg〖max〗_k q_ik.

Intuitively, this is a process of rearranging the latent space to align the “soft probability q calculated by the model” with the “more confident target p.” As a result, cluster boundaries become sharper, with similar patterns denser and different patterns further apart. When learning converges, the final label for each sample is set to kˆ_i = arg〖max〗_k q_ik.

**Figure 1:**
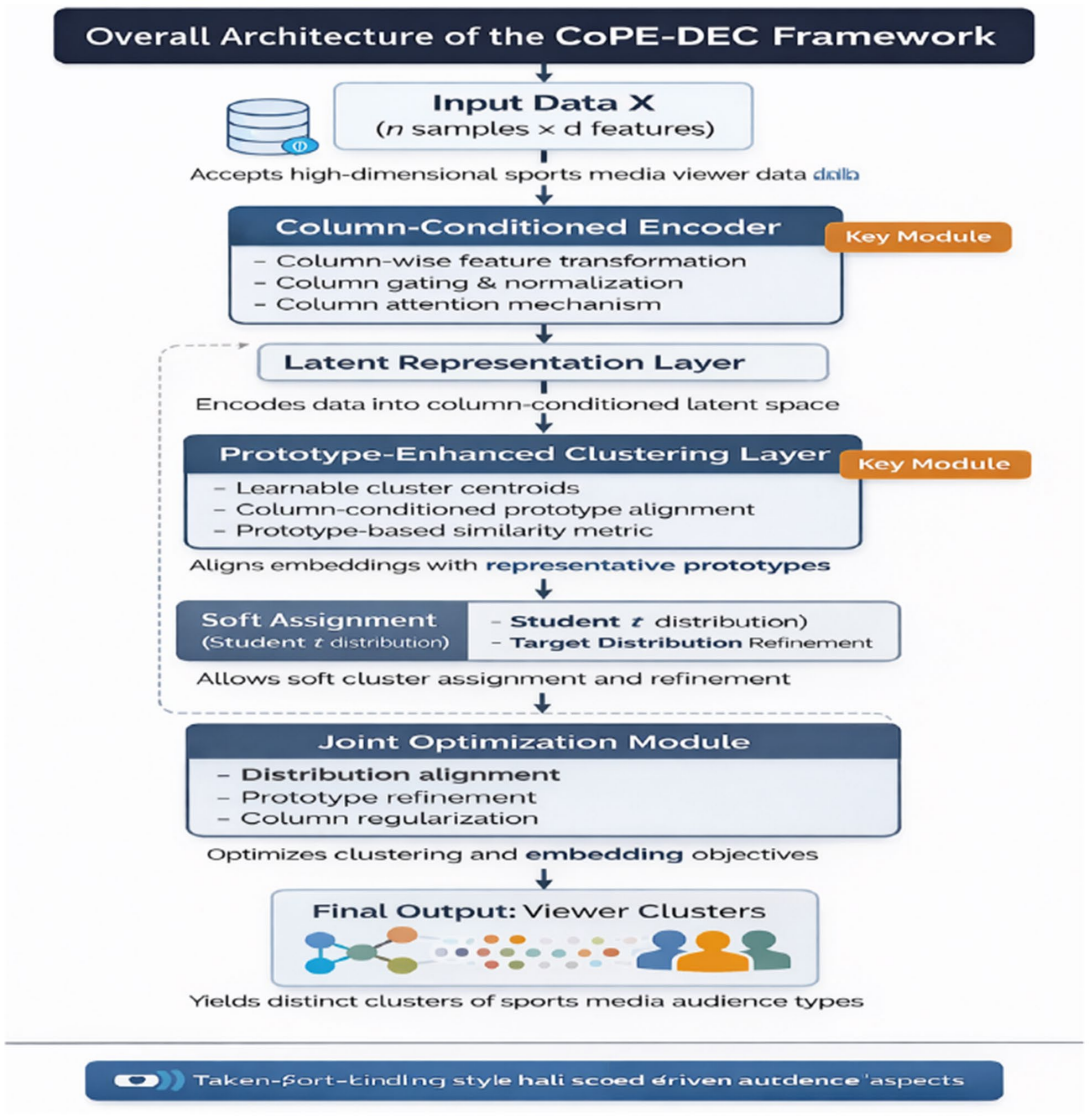
Overall architecture of the CoPE-DEC framework.

The production output merges these final labels into a single variable in the original table, and provides the upper-level items of the lift(k,c,v) = Pr (v∣k)/Pr (v) to provide the response defining the clusters at the column-category level. Quality assessment is based on intrinsic metrics (Silhouette–Hamming, Davies–Bouldin, Calinski–Harabasz), and the number of clusters is kept at least 3, and a reasonable candidate set (e.g., 3–7) is iterated over and the lowest Davies–Bouldin index is selected (Firman et al., 2023). Starting from these initial centers, Deep Embedded Clustering (DEC) is applied to fine-tune the boundaries. DEC jointly updates the encoder and centers by reducing the KL divergence to align the soft assignments computed with the Student-t kernel with the target distribution. As a result, the latent space is rearranged so that similar response patterns are closer together and different patterns are further apart, resulting in the final clusters (Figure 1).

### 4.4 Data preprocessing and augmentation

To ensure reproducibility and methodological rigor, the data preprocessing and augmentation procedures were explicitly defined and systematically applied prior to model training. First, identifier-like variables (e.g., response IDs, timestamps, or metadata not related to viewer behavior) were removed, as these features do not contribute meaningful semantic information and may introduce spurious correlations. In addition, categorical variables with extremely low frequency—defined as categories appearing in fewer than a predefined threshold of samples—were excluded to mitigate sparsity and reduce noise in the representation space.

For categorical features with sparse response distributions, a category consolidation rule was applied. Specifically, infrequent categories were grouped into an “other” class to preserve statistical reliability while maintaining semantic coherence. This procedure prevented the fragmentation of category-specific embeddings and ensured stable learning during column-conditioned encoding. All remaining features were encoded using a column-aware encoding strategy designed to preserve feature-level semantics. Each feature column was independently transformed according to its data type, ensuring that numerical and categorical variables retained their intrinsic meaning prior to projection into the shared latent space. This design aligns with the column-conditioned embedding mechanism employed in the proposed framework, enabling meaningful correspondence between input variables and learned representations.

To enhance model robustness and prevent overfitting, data augmentation strategies were incorporated during training. Specifically, a column-wise masking strategy was applied by randomly masking a subset of feature values within each batch, encouraging the model to learn redundant and robust representations. Additionally, a within-batch swapping strategy was employed, in which feature values were exchanged among samples within the same batch under controlled conditions. This augmentation approach increases data diversity while preserving the underlying marginal distributions and column-level semantics. Collectively, these preprocessing and augmentation steps ensure that the input data are semantically consistent, statistically stable, and suitable for deep clustering. By explicitly documenting each stage of the preprocessing pipeline, the revised manuscript supports full experimental reproducibility and strengthens the scientific validity of the proposed analysis.

### 4.5 Comparison with state-of-the-art (SOTA) deep clustering methods

While the baseline methods employed in this study were deliberately selected to ensure interpretability and compatibility with categorical, survey-based sports media data, we acknowledge the importance of situating the proposed framework within the broader landscape of recent state-of-the-art (SOTA) deep clustering research. Recent advances in deep clustering—particularly contrastive clustering, self-supervised representation learning, and transformer-based frameworks—have demonstrated remarkable performance on large-scale vision, text, and multimodal benchmarks (Caron et al., 2021; Li et al., 2023; Zhou et al., 2022). These approaches primarily focus on maximizing representation quality and clustering accuracy through end-to-end learning from high-dimensional, homogeneous, and often continuous input data.

However, the direct applicability of such SOTA models to column-structured, semantically heterogeneous survey data remains limited. Many recent frameworks implicitly assume feature homogeneity, spatial or temporal continuity, or modality-aligned embeddings, which are rarely satisfied in audience survey datasets composed of discrete, conceptually independent items (Xu et al., 2022; Abid et al., 2021). Moreover, end-to-end representation learning without explicit variable-level conditioning can obscure item semantics, thereby constraining interpretability—an essential requirement in human–computer interaction (HCI)–oriented audience research. To address this methodological gap, the revised manuscript clarifies the rationale for baseline selection by emphasizing data-type compatibility, semantic transparency, and interpretability requirements intrinsic to sports media viewer analysis. In contrast to many SOTA approaches that treat clustering accuracy as a primary objective, the proposed Column-conditioned Prototype-Enhanced Deep Embedded Clustering (CoPE-DEC) framework is explicitly designed to support human-centered analytical goals, where preserving item-level meaning and enabling theoretically grounded interpretation of viewer typologies are equally critical.

In addition, we introduce a conceptual comparison between CoPE-DEC and recent SOTA deep clustering frameworks, highlighting complementary strengths and inherent trade-offs. While contemporary models excel at learning abstract representations from unstructured data (Caron et al., 2021; Van Gansbeke et al., 2021), CoPE-DEC introduces several methodological innovations tailored to structured audience data: (1) column-conditioned embeddings that preserve feature-specific semantics, (2) prototype-aligned clustering mechanisms that enhance interpretability and stability, and (3) joint optimization strategies that balance representation learning with cluster refinement under semantic constraints. These design principles reflect a deliberate emphasis on HCI-aligned explainability and analytical robustness, rather than on marginal gains in clustering accuracy alone. Accordingly, the novelty of CoPE-DEC lies not in outperforming existing methods on generic benchmarks, but in providing a domain-adaptive, interpretable, and HCI-oriented clustering framework capable of uncovering latent viewer typologies within complex sports media environments. This positioning clarifies the contribution of the proposed method within contemporary deep clustering research, particularly for applications where semantic fidelity and interpretability are paramount.

Finally, to further strengthen empirical validation, future research will incorporate systematic benchmarking against emerging SOTA clustering models under comparable experimental conditions, including matched data structures, evaluation criteria, and interpretability constraints. Such extensions will enable a more comprehensive assessment of performance trade-offs and support the continued methodological refinement of AI-driven audience analysis frameworks in sports media and HCI research.

## 5 Results

In this study, the proposed CoPE-DEC model was compared with three alternative embedding-based clustering approaches to evaluate the performance of the final clusters. The baseline embedding models included Truncated SVD, Multiple Correspondence Analysis (MCA), and UMAP. For all models, K-means clustering was applied to the re-sulting embeddings to generate cluster assignments and facilitate comparison.

For the SVD embedding, a sparse matrix was constructed using one-hot encoding (Peiliang, 1998). Truncated SVD (e.g., d = 32, random_state = 42) was then fit to the entire dataset to obtain linear low-dimensional embeddings. K-means clustering (K = 3, k-means++, n_init = 20, fixed seed) was applied to this embedding space to assign cluster labels. Evaluation metrics, including Silhouette (Hamming), Davies–Bouldin Index (DBI), and Ca-linski Harabasz (CH) scores, were computed in the one-hot space to ensure consistency. Cluster size, imbalance, and Lift (Top-3) were also reported. For visualization, the same seed was used to project embeddings into 2D using PCA or UMAP.

In the MCA embedding, all survey items were treated as categorical, and the original responses were used to fit the MCA model, yielding factor scores (embeddings) for each respondent (Kareem et al., 2012). K-means clustering was then applied to the MCA embed-ding space to determine cluster labels. To maintain comparability, Silhouette (Hamming), DBI, CH, cluster size/imbalance, and Lift (Top-3) were calculated in the one-hot space. Visualization of the MCA embeddings was performed by projecting to 2D using PCA, dis-playing results as points (left panel) or points with centroids and convex hulls (right panel).

For the UMAP embedding, the dataset was first transformed into one-hot format, and UMAP was fit to generate nonlinear embeddings (Allaoui et al., 2020). K-means clustering was subsequently applied to the UMAP embeddings to obtain cluster labels. Evaluation followed the same procedure as the previous models, with metrics computed in the one-hot space and cluster size, imbalance, and Lift (Top-3) reported. For visualization consistency, a single 2D coordinate projection was generated for all models, with identical colors and legends applied to left and right panels.

As a result, the results of each of the four models were derived as shown in Table 1.

**Table 1:** Comparison of clustering results of models.

Model	Silhouette (Hamming)	Davies–Bouldin (one-hot)	Calinski–Harabasz (one-hot)	Size imbalance ratio
CoPE-DEC	0.2133	1.9498	84.8672	1.1014
SVD	0.284149	2.375365	87.5309	1.095335
MCA	0.334461	1.613688	75.461597	1.922921
UMAP	0.253339	2.513742	79.098446	1.265720

Therefore, cluster extraction was performed based on the finally selected CoPE-DEC model, and the results for over-represented categories were as shown in Table 2. When de-riving over-represented categories, it is a value that indicates which category appears frequently in a specific cluster, and this can be explained by Equation 14 (Premji et al., 2010). In Equation 14, #(c = v in k) represents the number of samples in cluster k where the variable c has the value v, and #(k) represents the total number of samples in k. And N represents the total number of samples in the entire data. Therefore, it is the intra-cluster ratio/overall ratio, where # represents the count of the corresponding denominator and numerator, respectively.


(14) $$\text{Lift}(c=v,k)=\frac{P(c=v|\text{cluster}=k)}{P(c=v)}=\frac{\frac{\#(c=v\;\text{in}\;k)}{\#(k)}}{\frac{\#(c=v)}{N}}$$

**Table 2:** Cope—clustering element results of DEC.

Cluster	*n*	Mean silhouette	Over-represented categories (top 3)
1	16.63%	0.688	Personal values = 3 (×3.817)
			Physical values = 3 (×3.120)
			Aesthetic values = 3 (×3.068)
			Economic values = 3 (×3.054)
2	30.02%	0.467	Media information = 5 (×3.275)
			Experiential tourism = 5 (×3.142)
			Direct participation = 5 (×3.086)
3	53.35%	−0.077	Emotional attitude = 1 (×1.875)
			Social attitude = 2 (×1.875)
			Positive attitude = 1 (×1.875)

## 6 Discussion

**Figure 2:**
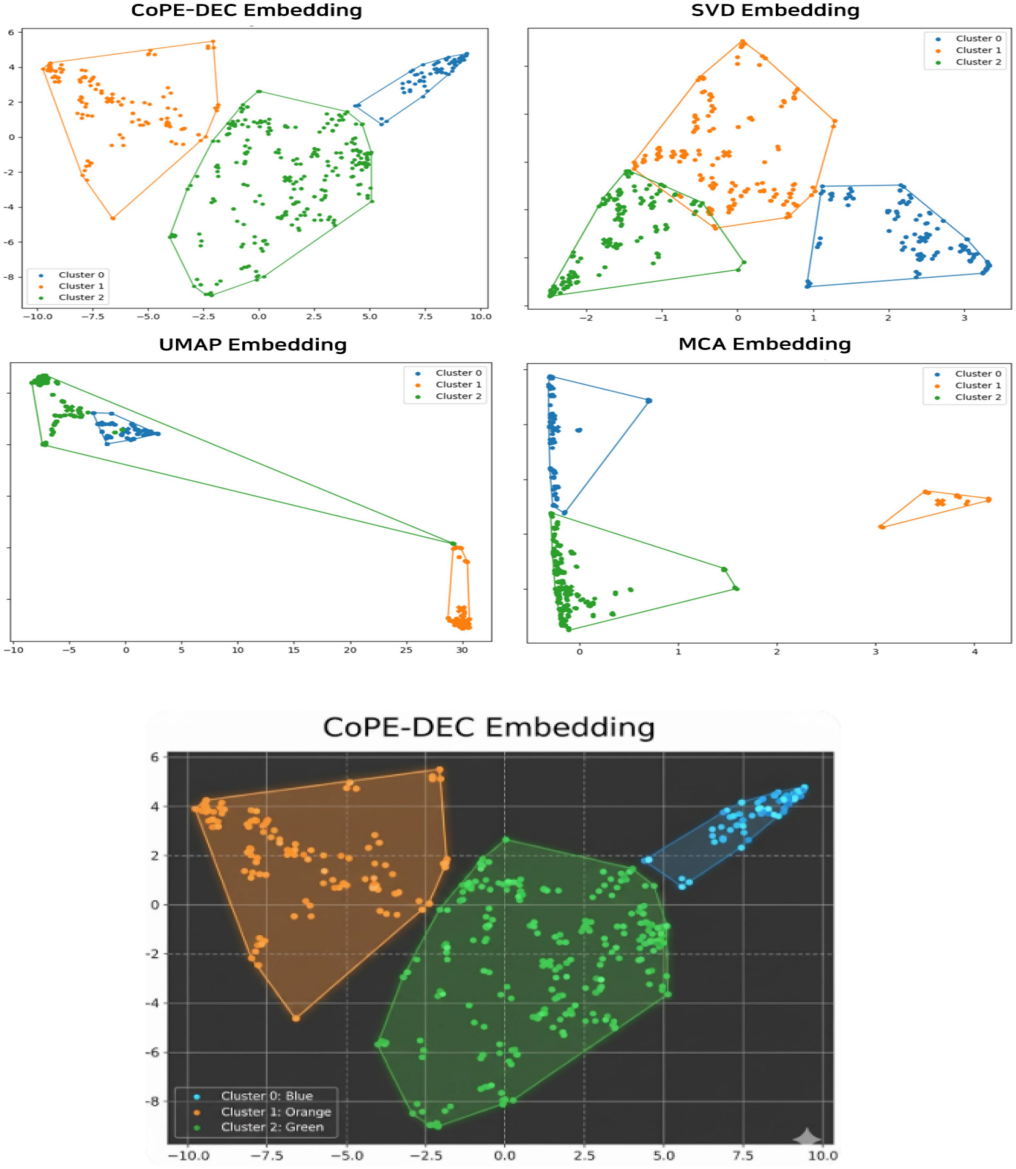
Clustering results of models.

This study aimed to identify latent viewer typologies in AI-driven sports media environments by applying the Column-conditioned Prototype-Enhanced Deep Embedded Clustering (CoPE-DEC) framework from a human–computer interaction (HCI) perspective. Extending beyond descriptive segmentation, the revised discussion provides an attribution-based interpretation of cluster formation by linking observed audience patterns to underlying HCI mechanisms embedded in contemporary digital sports media ecosystems (Figure 2 and Table 3).

**Table 3:** Clustering element features of CoPE-DEC embedding.

Cluster	Pattern features	Key elements
1	Pursuing sports values	Personal values: Improving concentration through sports viewing, fostering teamwork through sports viewing.
		Physical values: Motivating health, exercising, and vicarious satisfaction through sports viewing.
		Aesthetic values: Fascination with sports movements, artistic gestures in sports.
		Affinity values: Admiration for athletes featured in the media, admiration for the financial success of professional athletes.
2	Pursuing sports consumption culture	Media information: Prefers sports broadcasts, prefers sports broadcasts over entertainment programs, prefers online sports broadcasts and videos, and regularly watches sports of interest.
		Experiential tourism: Motivates people to experience sports through watching sports, participates in sports tourism products.
		Direct participation: Regularly participates in sports activities through watching sports, acquires sports skills through media, and pursues consumption of sports equipment.
3	Pursuing sports attitudes	Social attitudes: Watching sports helps with social adaptation, fosters new relationships, and enhances group solidarity.
		Emotional attitudes: Watching sports provides a stress-relieving experience, promotes psychological stability, and engaging in sports through watching sports fosters a healthy competitive spirit.
		Positive attitudes: Watching sports fosters a positive outlook on life overall, and a positive mindset due to stress relief.

### 6.1 Attributional interpretation of viewer clusters through HCI mechanisms

The emergence of three distinct viewer clusters can be explained by differentiated interaction affordances, psychological engagement mechanisms, and media-mediated social processes inherent in modern sports media platforms. In particular, immersion, parasocial interaction, and interface-mediated participation function as core explanatory mechanisms shaping viewer behavior and orientation.

#### 6.1.1 Cluster 1: sports value orientation and immersive cognitive engagement

Viewers classified under the “Sports Value Orientation” cluster demonstrated heightened concentration, aesthetic appreciation, admiration for athletes, and intrinsic motivation toward health and physical activity. These patterns can be attributed to deep immersive engagement, a central concept in HCI and media psychology. Advanced broadcast technologies—such as high-definition visuals, slow-motion replays, and data-enhanced commentary—facilitate cognitive absorption and attentional focus, enabling viewers to process sports content not merely as entertainment but as meaningful symbolic experiences (Shin and Biocca, 2018; Riva et al., 2022). From an attributional perspective, repeated exposure to immersive sports media reinforces internalized sports values by aligning sensory stimulation with cognitive and emotional appraisal processes. Recent studies suggest that immersive sports media experiences foster long-term value internalization, influencing physical activity intention, moral evaluation, and aesthetic judgment, particularly among digitally native audiences (Wei et al., 2025; Zhang and Zhang, 2023). However, commercialization and algorithm-driven highlight curation may simultaneously reshape value hierarchies by prioritizing spectacle and celebrity narratives over educational or ethical dimensions of sport (Gantz and Wenner, 2020; Lee et al., 2020). Cluster 1 thus reflects a tension between intrinsic sports values and mediated representations shaped by platform design.

#### 6.1.2 Cluster 2: sports consumption culture and interface-mediated participation

The “Sports Consumption Culture” cluster is characterized by frequent media consumption, sports-related tourism, merchandize purchases, and skill acquisition through digital platforms. This pattern can be causally linked to interface-mediated participation mechanisms, wherein platform architectures actively encourage transactional and experiential engagement. Features such as subscription models, recommendation algorithms, in-stream advertising, and integrated e-commerce transform viewers into active participants within a sports consumption ecosystem (Trail et al., 2021; Ratten, 2021). HCI research emphasizes that interfaces do not merely transmit content but structure user behavior by shaping choice architectures and participation pathways (Norman, 2013; Sundar, 2020). In this context, sports media platforms function as consumption orchestrators, lowering participation barriers and reinforcing habitual engagement cycles. The attribution of Cluster 2’s behavior lies in the synergistic interaction between user agency and system-level affordances, which collectively normalize continuous consumption and experiential diversification. This cluster exemplifies how sports fandom evolves into a cultural practice sustained by digital infrastructures rather than by event-centric attendance alone (Popp et al., 2019).

#### 6.1.3 Cluster 3: sports attitude orientation and parasocial–social integration

The “Sports Attitude Orientation” cluster reflects social adaptation, emotional regulation, stress relief, and group solidarity derived from sports viewing. These outcomes can be explained through parasocial interaction and social presence mechanisms, which are increasingly amplified in interactive sports media environments. Real-time chat, social media integration, and influencer-style commentary foster perceived intimacy and social connectedness, enabling viewers to experience sports as shared emotional events (Lim et al., 2024; Tukachinsky, 2021). From an HCI standpoint, these mechanisms reduce psychological distance and promote affective bonding, positioning sports media as a tool for emotional coping and identity reinforcement. Empirical evidence suggests that such digitally mediated social engagement enhances wellbeing, resilience, and sustained participation, particularly in post-pandemic media consumption contexts (Filo et al., 2015; Kim and Hwang, 2019). Cluster 3 thus represents an affective–social orientation shaped by the convergence of parasocial cues and community-based interaction interfaces.

### 6.2 Broader implications for HCI and the sports media industry

Collectively, the findings illustrate that contemporary sports media consumption is not driven by a single motivational logic but by distinct HCI-mediated pathways that produce differentiated viewer typologies. The integration of AI-driven clustering with HCI theory enables a more nuanced understanding of how interface design, interaction patterns, and psychological mechanisms jointly shape audience behavior. Practically, these insights have significant implications for sports media strategy. Content personalization, interface customization, and engagement design can be optimized by aligning platform features with the dominant HCI mechanisms associated with each viewer type. Moreover, the application of HCI extends beyond media consumption to athlete training, performance analysis, and fan engagement. Emerging technologies—such as AR/VR/MR, wearable sensors, and real-time feedback systems—facilitate immersive training environments, data-driven coaching, and enhanced fan experiences, reinforcing the convergence of performance analytics and user-centered design (Funk et al., 2018; Gürses et al., 2020; Riva et al., 2022).

### 6.3 Theoretical contributions and future directions

The present study contributes to sports media research by demonstrating how HCI-aligned deep clustering frameworks can move beyond descriptive segmentation toward explanatory audience modeling. By attributing cluster formation to specific interaction mechanisms, this study advances theoretical integration between AI-based audience analytics and HCI theory. Future research should further investigate causal pathways through longitudinal designs, multimodal data integration, and experimental manipulation of interface features. Additionally, benchmarking CoPE-DEC against emerging SOTA models under comparable conditions will strengthen generalizability while preserving interpretability and domain relevance.

## 7 Conclusion

This study proposed and empirically validated an HCI-oriented deep clustering framework—Column-conditioned Prototype-Enhanced Deep Embedded Clustering (CoPE-DEC)—to identify latent viewer typologies within contemporary AI-driven sports media environments. By moving beyond conventional descriptive segmentation, the study demonstrated that sports media audiences can be meaningfully classified according to distinct interaction-driven mechanisms that reflect how users cognitively, emotionally, and socially engage with digital sports content.

The findings revealed three interpretable viewer clusters—Sports Value Orientation, Sports Consumption Culture, and Sports Attitude Orientation—each shaped by different HCI-mediated processes, including immersive cognitive engagement, interface-mediated participation, and parasocial–social integration. Importantly, these clusters did not merely represent differences in viewing frequency or preference, but reflected deeper attributional mechanisms through which digital interfaces, media affordances, and psychological engagement jointly structure sports media consumption. This supports the argument that audience heterogeneity in digital sports media is fundamentally interactional rather than purely demographic or behavioral.

From a theoretical perspective, this study contributes to the literature by bridging AI-based audience analytics with human–computer interaction theory. While prior sports media research has largely treated segmentation as an outcome-oriented classification task, the present study reframes viewer typologies as emergent results of interaction mechanisms embedded in platform design and media systems. In doing so, it advances a mechanism-based understanding of sports media consumption that integrates immersion theory, parasocial interaction, and interface affordance theory within a unified analytical framework.

Methodologically, the study extends deep embedded clustering research by introducing a column-conditioned and prototype-aligned architecture tailored to structured, semantically heterogeneous survey data. Unlike many state-of-the-art deep clustering models optimized for unstructured or homogeneous inputs, CoPE-DEC prioritizes interpretability, semantic alignment, and stability—features that are critical for HCI-driven audience analysis and applied sports media research. This positions CoPE-DEC as a domain-adaptive alternative to accuracy-centered clustering approaches, particularly in contexts where explainability and human-centered interpretation are essential.

From a practical standpoint, the results offer actionable insights for the sports media industry. Understanding viewer typologies through HCI mechanisms enables media organizations to design differentiated content strategies, personalized interfaces, and engagement pathways aligned with specific audience orientations. Moreover, the implications of this framework extend beyond media consumption to adjacent domains, including fan engagement, athlete training, performance analytics, and immersive experience design, where HCI technologies such as AR/VR/MR, wearables, and real-time feedback systems are increasingly influential.

Despite these contributions, several limitations should be acknowledged. The cross-sectional nature of the data constrains causal inference, and the reliance on self-reported survey measures may not fully capture dynamic interaction behaviors. Future research should therefore employ longitudinal designs, multimodal data sources, and experimental manipulation of interface features to more rigorously test causal pathways. Additionally, systematic benchmarking against emerging state-of-the-art deep clustering models under matched conditions will further clarify performance trade-offs and enhance generalizability.

In conclusion, this study demonstrates that integrating deep learning with an HCI perspective enables a more explanatory, interpretable, and human-centered understanding of sports media audiences. By revealing how interaction mechanisms shape distinct viewer typologies, the proposed CoPE-DEC framework offers both a methodological advancement and a theoretical foundation for future AI-driven audience research in complex digital media environments.

## Data Availability

The raw data supporting the conclusions of this article will be made available by the authors upon reasonable request.
